# Potentially inappropriate medication in the elderly in Germany: an economic appraisal of the PRISCUS list

**DOI:** 10.1186/s12913-016-1366-x

**Published:** 2016-04-01

**Authors:** Katharina Pohl-Dernick, Florian Meier, Renke Maas, Oliver Schöffski, Martin Emmert

**Affiliations:** Chair of Health Management, Institute of Management (IFM), Friedrich-Alexander-Universität Erlangen-Nürnberg (FAU), Lange Gasse 20, 90403 Nuremberg, Germany; Institute of Experimental and Clinical Pharmacology and Toxicology, Friedrich-Alexander-Universität Erlangen-Nürnberg (FAU), Fahrstr. 17, 91054 Erlangen, Germany; Junior Professor for Health Services Management, Institute of Management (IFM), Friedrich-Alexander-Universität Erlangen-Nürnberg (FAU), Lange Gasse 20, 90403 Nuremberg, Germany

**Keywords:** Potentially inappropriate medication, Elderly, Drug Reimbursement, PRISCUS, Reimbursement costs

## Abstract

**Background:**

Several lists of potentially inappropriate medication (PIM) for elderly patients have been developed worldwide in recent years. Those lists intend to reduce prescriptions of drugs that carry an unnecessarily high risk of adverse drug events in elderly patients. In 2010, an expert panel published the PRISCUS list for the German drug market. This study calculates the amount of drug reimbursement for PIM in Germany and potential cost effects from the perspective of statutory health insurance when these are replaced by the substitutes recommended by the PRISCUS list.

**Methods:**

Register-based data for the 30 top-selling drugs on the PRISCUS list in 2009 for patients greater than or equal to 65 years of age were provided by the Scientific Institute of the German Local Health Care Fund. We calculated the percentage of sales and defined daily doses for patients greater than or equal to 65 years of age compared with the total statutory health insurance population. Reimbursement costs for the recommended substitutions were estimated by considering different scenarios.

**Results:**

In 2009, drug reimbursement for the 30 top-selling PIM prescribed to patients greater than or equal to 65 years of age were calculated to be €305.7 million. Prescribing the recommended substitution medication instead of PIM would lead to an increased total reimbursement cost for the German health care system ranging between from €325.9 million to €810.0 million.

**Conclusions:**

The results show that the substitution of PIM by medication deemed to be more appropriate for the elderly comes along with additional costs. Consequently, there is no short-term incentive for doing so from a payer perspective. Future studies have to consider the long-term effects and other sectors.

## Background

Potentially inappropriate medication (PIM) can influence the safety of elderly patients’ drug therapy. Several international studies have shown that PIM might lead to higher morbidity and mortality as well as it contributes to adverse drug events (ADE) and hospital admissions [1–3]. Consequently, PIM might have an impact on the health and quality of life of elderly people. Thus, a need has emerged for the systematic identification and description of inadequate pharmacotherapy for older patients. Different classification systems of critical drugs have been developed in many countries to support physicians in clinical practice such as the Beers criteria in the US [4], McLeod in Canada [5], and Laroche in France [6]. The prevalence of PIM is calculated between 13 % and 43 %, depending on the tool used and the study setting [7, 8].

In Germany, the Federal Ministry of Health initiated an evaluation of PIM in 2008/2009 considering the country-specific characteristics of the pharmaceutical market. Details of the development process have been described elsewhere [9]. The result was the so-called PRISCUS list, which comprises 83 agents classified as PIM. Altogether, 64 agents are listed on one or more international PIM lists, 12 agents are only available in Germany and seven are solely classified as PIM in Germany [9]. In contrast to former frameworks, the PRISCUS list is more than a listing of critical agents for elderly patients. Additional information about the adequate substitution of PIM is provided [9, 10]. Several studies evaluate PIM in Germany and have determined the prevalence to be between 18 % and 43 %, depending on the setting and patient group [11–14]. However, the social and economic burden has not been addressed so far.

The aim of this investigation was to analyse the reimbursement costs of PIM according to the PRISCUS list and to evaluate the economic effects of a recommended substitution from the perspective of SHI in Germany. As the PRISCUS list was explicitly developed for the German pharmaceutical market, we will use it to detect PIM in the German SHI population. We provide information about (a) the proportion of agents on the PRISCUS list prescribed to elderly people ≥65 years of age, (b) the reimbursement costs of PIM from the perspective of SHI and (c) the potential costs of the recommended substitution medication.

## Methods

The methodology of the study consists of three components. First, anonymised medication data on patients ≥65 years of age were obtained and enriched with information about the entire SHI population. Second, costs for the prescription of surrogates were calculated based on different scenarios. Third, we conducted a literature research to identify international studies for comparison with our results.

### Data source

The underlying anonymised aggregated prescription data were provided by the Scientific Institute (WIdO) of the German Local Health Care Fund (AOK). It was founded in 1976 and has the function to carry out research projects aimed at ensuring high quality and economic health care for the community of the Local Health Care Funds [15]. The WIdO has a prescription register of all drugs prescribed to patients in German SHI, excluding drugs dispensed at hospitals, over-the-counter (OTC) compounds, and prescriptions of private health insurance. Our study material is a register extraction of PRISCUS agents solely given to patients aged ≥65 years in 2009. The WIdO provided the sales volume, number of prescriptions, and the prescribed defined daily dose (DDD) for the top 30 PRISCUS agents with the highest sales volume. Additionally, the total sales volume, the total number of prescriptions, and the total amount of DDDs for all 83 agents on the PRISCUS list for the patient group ≥65 years of age were provided.

The data set was enhanced with additional information from the pharmaceutical prescription report from 2010 (PPR) [16]. The PPR is an annual publication by the WIdO about the 3000 most frequently prescribed drugs to the SHI population in Germany. Therefore, the provided data set and the enriched data have the same origin. First, we extracted the sales volume, prescriptions, DDDs, and costs per DDD of all PRISCUS agents for the entire SHI population from the PPR. Second, based on the enhanced data set, we calculated the share of patients ≥65 years of age in the entire SHI population for sales volume, prescriptions, and DDDs.

### Scenarios

To calculate the potential costs of the recommended drug substitutions, we first derived the costs per DDD for the recommended surrogates from the PPR. In the first case, if a specifically drug was named, the costs per DDD were directly taken from the PPR. In the second case, if a drug group was recommended as an adequate alternative, a weighted average based on the costs per DDD and the prescribed annual DDDs was calculated. For example, the recommended alternatives on the PRISCUS list for indometacin are (1) paracetamol, (2) weak opioids (tramadol and codeine), (3) metamizol (after careful consideration of the risks and the benefits) and (4) "weaker" NSAIDs (e.g., ibuprofen) [9]. For (1) paracetamol and (3) metamizol, the costs per DDD were taken from the PPR (€0.49/DDD and €1.51/DDD). For (2) weak opioids, tramadol and codeine are explicitly recommended. Thus, the costs per DDD for these two agents were also taken from the PPR (€1.19/DDD and €2.65/DDD). By contrast, for (4) “weaker” NSAIDs, ibuprofen is only one example of a possible substitution. Thus, the costs per DDD for the whole group of "weaker" NSAIDs were calculated (€0.56/DDD).

In a second step, the reimbursement costs for an adjusted drug therapy was estimated under the consideration of the recommended surrogate according to the PRISCUS list. We assumed that the amount of DDDs for the recommended surrogates would not differ from the actual prescribed DDDs of PIM. To calculate the economic impact, the costs per DDD for the proposed substitution were multiplied by the prescribed amount of DDDs for patients ≥65 years of age. As several drugs or drug groups are recommended in some cases, a range of costs per DDD for surrogates had to be considered. Thus, we performed three different scenarios. Besides the cheapest (Scenario 1) and the most expensive surrogate (Scenario 2), we also calculated the mean costs per DDD of the proposed alternatives (Scenario 3) and the corresponding costs of the prescription.

### Literature review

Two systematic literature reviews were conducted to compare the results with national and international studies. The first one consisted of studies dealing with the prevalence of PIM in Germany measured by applying the PRISCUS list. The second one focused on international studies analysing reimbursement costs. We carried out two Medline searches on the 2^nd^ of November 2014. In order to identify the prevalence of PIM in Germany, we combined 18 synonyms for PIM, such as potentially inappropriate medication, inappropriate prescribing, and inappropriate practice in prescribing with “PRISCUS”. For the identification of studies of the costs of PIM, we combined the keywords cost OR costs OR cost analysis OR cost analyses OR drug reimbursement OR drug reimbursements OR economic burden OR economic evaluation and elder* OR old* OR geriatric* OR senior OR aged OR aging with the same words on PIM as in the search on prevalence. The complete search history for both searches can be obtained from the first author upon request. The search was not limited with regard to language and timeframe. Identified studies were searched by reviewing the references as well. For search 1, only studies of the prevalence of PIM in Germany were included. For search 2, papers were included if they examined the nationwide costs of PIM on an annual basis. Papers only examining the prevalence or the applicability of tools to identify PIM were excluded.

## Results

In 2009, the total expenditure for all 83 agents on the PRISCUS list was €387.8 million and the reimbursement costs of the 30 top-selling agents amounted to €305.7 million (78.8 %). The highest reimbursement costs were caused by solifenacin, with €32.5 million, followed by etoricoxib (€30.6 million), and zopiclone (€21.5 million), as displayed in Table 1. Those three agents accounted for approximately 20 % of the total sales volume. Compared with the entire SHI population in Germany, the total sales for the 30 top-selling agents were €505.5 million and €562.7 million for all 83 agents PRISCUS agents, respectively. Thus, 54.3 % and 37.4 % of sales can be assigned to patients ≥65 years of age.

**Table 1:** 30 top-selling agents on PRISCUS list (Prices in €, Reference year 2009)

			Aged 65 and above				Entire SHI population				Ratio aged ≥65 and entire SHI		
No.	ATC-Code	Agent	DDD (in Mio.) ≥65 years	Prescriptions ≥65 (in Mio.)	Sales ≥65 (in Mio. €)	Costs per DDD ≥65	Total DDD (in Mio)	Total prescriptions (in Mio)	Total sales (in Mio. €)	Costs per DDD	DDD	Prescriptions	Sales
1	C02CA04	Doxazosin	51.4	0.56	19.5	0.38 €	60.2	0.81	28.1	0.38 €	85.38 %	69.47 %	69.37 %
2	N06AA09	Amitriptyline	45.7	1.13	19.8	0.43 €	94.5	2.28	40.5	0.43 €	48.36 %	49.66 %	48.88 %
3	C07AA07	Sotalol	34.0	0.53	10.2	0.30 €	36.6	0.68	13.0	0.30 €	92.90 %	78.39 %	78.56 %
4	C01AA02	Acetyldigoxin	32.5	0.93	11.7	0.36 €	36.4	1.05	13.3	0.36 €	89.29 %	88.62 %	88.06 %
5	N05CF01	Zopiclon^a^	28.0	1.44	21.5	0.77 €	47.3	2.62	38.7	0.79 €	59.20 %	54.87 %	55.56 %
6	M01AH05	Etoricoxib	25.2	0.50	30.6	1.21 €	51.7	1.07	62.4	1.21 €	48.74 %	46.61 %	49.04 %
7	N06AA12	Doxepin	24.2	0.77	13.1	0.54 €	54.2	1.68	29.1	0.52 €	44.65 %	45.71 %	45.07 %
8	G04BD08	Solifenacin	22.6	0.24	32.5	1.44 €	31.3	0.33	44.8	1.43 €	72.20 %	72.84 %	72.54 %
9	N05CF02	Zolpidem^a^	21.3	1.09	16.3	0.77 €	32.5	1.81	26.7	0.78 €	65.54 %	60.36 %	61.14 %
10	N06BX03	Piracetam	18.3	0.41	8.2	0.45 €	20.6	0.48	9.7	0.45 €	88.83 %	85.85 %	84.86 %
11	C04AD03	Pentoxifylline	15.0	0.35	7.2	0.48 €	17.7	0.57	11.0	0.54 €	84.75 %	61.29 %	65.61 %
12	N05BA08	Bromazepam	14.9	0.66	8.6	0.58 €	21.2	1.04	13.5	0.60 €	70.28 %	63.40 %	63.65 %
13	N06AA06	Trimipramine	14.6	0.49	10.8	0.74 €	31.6	1.12	24.5	0.73 €	46.20 %	43.66 %	44.17 %
14	N05BA01	Diazepam	13.1	0.53	6.0	0.46 €	31.0	1.30	15.3	0.47 €	42.26 %	40.71 %	39.16 %
15	N05CD06	Lormetazepam^a^	12.5	0.38	5.2	0.42 €	15.3	0.51	7.0	0.42 €	81.70 %	74.50 %	74.20 %
16	C02CA08	Terazosin	12.3	0.19	6.6	0.54 €	8.0	0.24	8.2	0.56 €	-^b^	-	-
17	C02AC01	Clonidine	11.7	0.33	5.9	0.50 €	17.7	0.71	13.5	0.34 €	66.10 %	46.72 %	43.86 %
18	C02LA01	Reserpine and Diuretics	11.3	0.12	4.7	0.42 €	13.1	0.14	5.5	0.42 €	86.26 %	88.82 %	85.45 %
19	C01BC04	Flecainide	9.7	0.22	11.4	1.18 €	14.1	0.34	17.5	1.17 €	68.79 %	64.05 %	65.30 %
20	G04BD04	Oxybutynin	9.3	0.30	8.8	0.95 €	11.3	0.45	15.1	0.95 €	82.30 %	66.64 %	58.14 %
21	M01AC06	Meloxicam	9.3	0.21	3.3	0.35 €	14.8	0.41	6.3	0.38 €	62.84 %	50.66 %	52.12 %
22	N06AB03	Fluoxetine	8.8	0.10	2.9	0.33 €	43.3	0.58	17.3	0.32 €	20.32 %	17.32 %	16.79 %
23	M01AB01	Indometacin	8.7	0.25	4.0	0.46 €	14.2	0.53	8.6	0.48 €	61.27 %	47.36 %	46.63 %
24	C01AA08	Metildigoxin	8.4	0.17	2.7	0.32 €	9.5	0.20	3.0	0.32 €	88.42 %	86.65 %	90.00 %
25	M03BX07	Tetrazepam	8.3	0.57	8.0	0.96 €	22.0	2.08	26.8	1.19 €	37.73 %	27.46 %	29.90 %
26	C04AX21	Naftidrofuryl	7.5	0.34	8.2	1.09 €	9.0	0.43	10.2	1.11 €	83.33 %	78.49 %	80.17 %
27	C08CA05	Nifedipine^a^	6.5	0.26	3.8	0.58 €	69.7	1.32	22.2	0.29 €	9.33 %	19.70 %	17.11 %
28	M01AB11	Acemetacin	6.4	0.16	4.9	0.77 €	9.8	0.28	8.3	0.80 €	65.31 %	56.34 %	58.76 %
29	M03BX01	Baclofen	6.0	0.26	5.2	0.87 €	17.1	0.66	16.1	0.93 €	35.09 %	39.12 %	32.31 %
30	J01XE01	Nitrofurantoin	5.4	0.26	4.1	0.76 €	8.7	0.43	6.7	0.85 €	62.07 %	59.99 %	61.45 %
Sum Top30			502.9	13.75	305.7		864.40	26.15	562.71		58.18 %	52.59 %	54.33 %
Sum all 83 agents			598.7	16.93	387.8		1,124.00	37.71	1,037.37		53.27 %	44.89 %	37.38 %

^a^PRISCUS agents which are potentially inappropriate at certain dose or release form

^b^The PPR does include the 3,000 top-selling drugs. Several traded products with terazosin as agent are sold less frequently and therefore not included. The published amount of DDD of terazosin is lower than the share of DDD taken by the elderlies. Consequently, calculation is misleading

Focusing on the number of PIM prescriptions to the elderly, there were 13.75 million prescriptions of the 30 top-selling PIM. For all PRISCUS agents, there were 16.93 million prescriptions to the elderly in 2009. Compared with the entire SHI population with 26.15 million prescriptions of the 30 top-selling PRISCUS agents and 31.71 million prescriptions of all 83 PRISCUS agents, 52.6 % and 44.9 % of the prescriptions were allocated to the elderly, respectively. Regarding all prescriptions to the entire SHI population (626.3 million in 2009), the elderly obtained 56 % (350.7 million) of them [16]. Consequently, the number of PIM prescriptions to the elderly compared with the total prescriptions to patients ≥65 years of age was 4.8 %.

The sum of DDDs for the 30 top-selling PRISCUS agents was 502.9 million DDDs and 598.3 million DDDs for all 83 agents. The leading agents were doxazosin (51.4 million DDDs), amitriptyline (45.7 million DDDs), and sotalol (34.0 million DDDs). In the entire SHI population, 864.40 million DDDs of the 30 top-selling PIM and 1,124.0 million DDDs of all PIM were prescribed. Thus, the majority of prescribed DDDs of the 30 top-selling PIM (58.2 %) may be assigned to patients for whom the drugs are potentially inadequate. Regarding all PIM on the PRISCUS list, 53.3 % of the DDDs for the entire SHI population were prescribed to the elderly.

Table 2 shows information on the costs and DDDs for PIM and the recommended surrogates on the PRISCUS list. When applying Scenario 1, the reimbursement costs for the 30 top-selling agents on the PRISCUS list would be €325.93 million and this would lead to higher total costs of €20.23 million (+6.6 %). For Scenario 2, the costs of medication would rise to €810.03 million and cause additional costs of €504.33 million (+165.0 %). By applying Scenario 3, the costs for the 30 top-selling drugs would rise by €124.76 million to €430.46 million (+40.8 %).

**Table 2:** Costs of surrogates for the elderly (Prices in €, Reference year 2009)

ATC-Code	Agent	Costs per DDD in €	DDD (in Mio.)	Surrogates according to PRISCUS list	Costs per DDD of surrogates in €					Total costs of surrogates in Mio. €		
					Min		Max		Mean	Min	Max	Mean
C02CA04	Doxazosin	0.38	51.4	Other antihypertensives: ACE inhibitors, angiotensin receptor blockers, diuretics, beta blocker, calcium channel blocker	ACE inhibitors	0.13	Angiotensin receptor blockers	1.08	0.23	6.64	55.51	23.73
N06AA09	Amitriptyline	0.43	45.7	SSRIs, mirtazapine	SSRIs	0.49	Mirtazapine	0.69	0.53	22.39	31.53	23.32
C07AA07	Sotalol	0.30	34.0	Beta blocker, amiodarone, propafenone	beta blocker	0.30	Amiodarone	0.72	0.77	10.09	24.48	21.69
C01AA02	Acetyldigoxin	0.36	32.5	Beta blocker, diuretics, ACE inhibitors	ACE inhibitors	0.13	Beta blocker	0.30	0.18	4.20	9.65	24.43
N05CF01	Zopiclon^a^	0.77	28.0	Valerian, sedative anti-depressants (trazodone, mianserin, mirtazapine), opipramol, low-potency neuroleptics (melperone & pipamperone)	Valerian	0.00	Low-potency neuroleptics	2.32	0.77	0.00	64.86	11.80
M01AH05	Etoricoxib	1.21	25.2	Paracetamol, weak opioids (codeine, tramadol), Metamizole, weak NSAID, antidepressants (long-acting tranquilizers, tricyclic antidepressants, other non-serotomergic monoamine oxidase inhibitors, SSRIs, other Selective reuptake inhibitors, other antidepressants), anticonvulsants (older anti-epileptics, newer anti-epileptics, neuromuscular blocking agents)	Paracetamol	0.49	Newer anti-epileptics	4.03	3.90	12.35	101.56	16.50
N06AA12	Doxepin	0.54	24.2	SSRIs, mirtazapine	SSRIs	0.49	Mirtazapine	0.69	0.53	11.86	16.70	12.94
G04BD08	Solifenacin	1.44	22.6	Trospium	Trospium	1.05	Trospium	1.05	1.05	23.73	23.73	5.89
N05CF02	Zolpidem^a^	0.77	21.3	Valerian, sedative anti-depressants (trazodone, mianserin, mirtazapine), opipramol, low-potency neuroleptics (melperone & pipamperone)	Valerian	0.00	Low-potency neuroleptics	2.32	0.77	0.00	49.34	2.95
N06BX03	Piracetam	0.45	18.3	ACE inhibitors (donepezil, galantamine, rivastigmine), Memantine	Memantine	3.86	Acetylcholinesterase inhibitors	4.25	4.11	70.64	77.78	7.80
C04AD03	Pentoxifylline	0.48	15.0	ACE inhibitors (donepezil, galantamine, rivastigmine), Memantine	Memantine	3.86	Acetylcholinesterase inhibitors	4.25	4.11	57.90	63.75	10.36
N05BA08	Bromazepam	0.58	14.9	Lorazepam, lormetazepam, short-acting benzodiazepine & benzodiazepine receptor agonists (zolpidem, zopiclone, brotizolam, triazolam, zaleplon), opipramol, mirtazapine, low-potency neuroleptics (melperone & pipamperone)	Lormetazepam	0.42	Low-potency neuroleptics	2.32	0.75	6.26	34.52	9.77
N06AA06	Trimipramine	0.74	14.6	SSRIs, mirtazapine	SSRI	0.49	Mirtazapine	0.69	00.53	7.15	10.07	11.23
N05BA01	Diazepam	0.46	13.1	Lorazepam, lormetazepam, short-acting benzodiazepine & benzodiazepine receptor agonists (zolpidem, zopiclone, brotizolam, triazolam, zaleplon), opipramol, mirtazapine, low-potency neuroleptics (melperone & pipamperone)	Lormetazepam	0.42	Low-potency neuroleptics	2.32	0.75	5.50	30.35	75.19
N05CD06	Lormetazepam^a^	0.42	12.5	Valerian, sedative anti-depressants (trazodone, mianserin, mirtazapine), opipramol, zolpidem, low-potency neuroleptics (melperone & pipamperone)	Valerian	0.00	Low-potency neuroleptics	2.32	0.78	0.00	28.96	30.82
C02CA08	Terazosin	0.54	12.3	Other antihypertensives: ACE inhibitors angiotensin receptor blockers, diuretics, beta blocker , calcium channel blocker (except nifedipine)	ACE inhibitors	0.13	Beta blocker	0.30	0.18	1.59	3.65	6.79
C02AC01	Clonidine	0.50	11.7	Other antihypertensives: ACE inhibitors, angiotensin receptor blockers, diuretics, beta blocker , calcium channel blocker	ACE inhibitors	0.13	Angiotensin receptor blockers	1.08	0.23	1.51	12.64	61.64
C02LA01	Reserpine and Diuretics	0.42	11.3	Other antihypertensives: ACE inhibitors, angiotensin receptor blockers, diuretics, beta blocker, calcium channel blocker (except nifedipine)	ACE inhibitors	0.13	Beta blocker	0.30	0.18	1.46	3.35	2.24
C01BC04	Flecainide	1.18	9.7	Beta blocker, amiodarone	Beta blocker	0.30	Amiodarone	0.72	0.30	2.88	6.98	9.87
G04BD04	Oxybutynin	0.95	9.3	Trospium	Trospium	1.05	Trospium	1.05	1.05	9.77	9.77	2.69
M01AC06	Meloxicam	0.35	9.3	Paracetamol, weak opioids (codeine, tramadol), metamizole, “weaker” NSAID	Paracetamol	0.49	Codeine	2.65	0.72	4.56	24.65	9.69
N06AB03	Fluoxetine	0.33	8.8	Other SSRIs (citalopram, paroxetine, escitalopram, sertraline, escitalopram), trazodone, mitrazapine	other SSRIs	0.51	Trazodone	1.44	0.56	4.50	12.67	8.71
M01AB01	Indometacin	0.46	8.7	Paracetamol, weak opioids (codeine, tramadol), metamizole, “weaker” NSAID	Paracetamol	0.49	Codeine	2.65	0.72	4.26	23.06	4.60
C01AA08	Metildigoxin	0.32	8.4	Beta blocker, diuretics	Diuretics	0.21	Beta blocker	0.30	0.26	1.76	2.49	2.06
M03BX07	Tetrazepam	0.96	8.3	Tolperisone, short-acting benzodiazepine & benzodiazepine receptor agonists (zolpidem, zopiclone, brotizolam, triazolam, zaleplon), intermediate-acting tranquilizers (bromazepan, oxazepam, lorazepam, alprazolam, buspirone)	intermediate-acting Tranquilizers	0.71	Tolperisone	1.55	0.82	5.89	12.87	12.59
C04AX21	Naftidrofuryl	1.09	7.5	ACE inhibitors (donepezil, galantamine, rivastigmine), memantine	Memantine	3.86	Acetylcholinesterase inhibitors	4.25	4.11	28.95	31.88	6.25
C08CA05	Nifedipine^a^	0.58	6.5	Other antihypertensives: ACE inhibitors, angiotensin receptor blockers, diuretics, beta blocker, calcium channel blocker (except nifedipine)	ACE inhibitors	0.13	Beta blocker	0.30	0.18	0.84	1.93	1.19
M01AB11	Acemetacin	0.77	6.4	Paracetamol, weak opioids (codeine, tramadol), metamizole, “weaker” NSAID	Paracetamol	0.49	Codeine	2.65	0.72	3.14	16.96	6.68
M03BX01	Baclofen	0.87	6.0	Tolperisone, tizanidine	Tizanidine	1.09	Tolperisone	1.55	1.45	6.54	9.30	4.91
J01XE01	Nitrofurantoin	0.76	5.4	Other antibiotics in accordance with antibiogramm: cephalosporins, cotrimoxazole, trimethoprim	Cotrimoxazole	1.77	Trimethoprim	2.79	2.33	9.56	15.07	2.16
Sum Top 30			502.9							325.93	810.03	430.46

^a^PRISCUS agents which are potentially inappropriate at certain dose or release form

Considering the results for Scenario 1, only 10 surrogates lead to increased reimbursement costs, whereas the substitution of the remaining 20 agents leads to a cost decrease. However, these 10 surrogates increase the costs by +200.0 % on average, whereas the decrease is -53.3 % on average. The cost driver in Scenario 1 is memantine (€3.86/DDD), a proposed surrogate for piracetam (€0.45/DDD), pentoxifyllin (€0.54/DDD), and naftidrofuryl (€1.11/DDD). The substitution of these three agents by memantine causes costs of €70.6 million, €57.9 million and €29.0 million, respectively. This results in a potential cost increase of €133.9 million (+467.0 %), from €23.6 million to €157.5 million.

In Scenario 2, 22 agents increase the costs by +289.0 % on average. The most cost-intensive surrogates are acetycholinesterase inhibitors (€4.25/DDD) – as proposed alternatives for piracetam, pentoxifyllin, and naftidrofuryl – and this would lead to increased reimbursement costs of €173.4 million (+635.0 %), from €23.6 million to €197.0 million. The other cost drivers in Scenario 2 are the recommended substitutes for etoricoxib (e.g., lamotrigine, oxcarbazepine, gabapentin). This substitution would raise reimbursement costs from €30.6 million to €101.56 million (+232.0 %). If indomethacin (0.46€/DDD) was substituted by codeine (2.65€/DDD), the cost of the medication would rise by +476.4 %. Only the substitution of eight agents would lead to a decrease in total reimbursement costs, by -27.5 %.

Considering the mean surrogate costs in Scenario 3, in 14 cases the substitution of the 30 top-selling agents would lead to a decrease in reimbursement costs by -37.9 % on average. However, these savings would be compensated for by the 16 remaining agents, which lead to an average increase of +160.7 %.

## Discussion

To our knowledge, this is the first study to assess the reimbursement costs of PIM according to the PRISCUS list in Germany from a SHI perspective. We conducted a comprehensive analysis based on prescription data from 2009 in order to depict the prevalence of PIM in the elderly in Germany and to quantify the economic impact of prescriptions as well as the substitution costs for the recommended medication.

### The prevalence of PIM in the elderly

Our search yielded eight studies dealing with the prevalence of PIM in Germany, according to the PRISCUS list [12–14, 17–21]. The present study is based on prescription data in the ambulant stetting for all patients ≥65 years of age in German SHI, and results in a prescription prevalence of 4.8 % in 2009. The investigations of Amann et al. and Schubert et al. are most comparable regarding the database. Amann et al. calculated a standardised prevalence of 28.3 % based on data on 800,000 insurees [17]. A similar study design was chosen by Schubert et al. [13]. They used claims data from German health insurance providers to calculate a prevalence of 22.0 %. However, they both analysed the prevalence at the patient level not at the prescription level. Therefore, the comparison is limited.

The six remaining studies calculated prevalence based on smaller patient samples and in different study settings. The results lay between 16.6 % in an emergency department [14] and 43 % in geriatric rehabilitation [12]. For patients in nursing homes, a prevalence of 31.2 % was calculated [18], while for elderly people living at home the prevalence was lower at 18 % [19], 28.4 % [20] and 29.0 % [21]. Thus, the prevalence of PIM in the elderly in Germany is between one quarter and one third , but this depends on the study setting, possible limitations concerning indication, data collection, and the consideration of the limitations of the PRISCUS list (dose dependency and release form).

### Economic assessment of PIM and the recommended substitution

A search of economic analyses showed that limited information is available. In total, 12 publications dealing with the costs of PIM were identified, but none of them considered German SHI. The studies were conducted in the US [22–26], Ireland [7, 27, 28], Japan [8], Finland [29], Saudi Arabia [30], and Switzerland [31]. They used different lists and mostly calculated the costs of PIM at the patient level [7, 8, 22, 25]. As our study addresses the annual economic relevance of PIM for SHI in Germany, only four studies from the US [24], Ireland [27, 28], and Finland [29] that addressed the national perspective are eligible for the comparison.

Bradley et al. analysed the PIM of patients >70 years of age according to the STOPP criteria in Northern Ireland, using data from the enhanced prescribing database of the National Health Service [27]. In total, costs of €6.1 million in 2009/2010 were calculated. This equals 5.38 % of total expenses on pharmaceuticals for people >70 years of age in Northern Ireland. Another study of Ireland by Cahir et al. published costs of €45.6 million for drug ingredients, tax, and pharmacy surcharges in 2007, representing 9 % of the overall expenditure on pharmaceuticals in people aged >70 years [28]. The calculation applying the STOPP criteria was based on the pharmacy claims of the National Shared Services Primary Care Reimbursement Service of the Health Service Executive. In the third study, Leikola et al. calculated reimbursement costs of €2.9 million for PIM medication to people >65 years of age in Finland in 2007, using the criteria of Fick and Beers [29]. This represents 0.7 % of total drug reimbursement for people aged >65 in Finland. The register-based cross-sectional study used data from Finland’s social insurance institution. The study from Fu et al., conducted in the US, calculated total health care expenditures of US$7.2 billion for community-dwelling patients exposed to PIM according to the criteria of Fick and Beers [24]. For medication, about 20 % of these costs were calculated. Consequently, costs of approximately US$1.44 billion were calculated for PIM in people >65 years of age in 2001 in the US.

In our study, the reimbursement costs of PIM were €387.8 million. Thus, the total reimbursement costs for PIM prescriptions in Germany are significantly higher than those in Finland, Ireland, and Northern Ireland, but lower than those in the US. The total sales of all proprietary mediclinical products in SHI were €28,499 million [16]. Thus, the proportion of PIM sales to elderly patients represents 1.36 %. However, a comparison of this number is hardly possible, as the studies from Finland, Ireland, and Northern Ireland used sales to elderly patients as the reference figure.

In general, the comparability of our results with other international studies is limited. First, different tools were used to detect PIM: Bradley et al. and Cahir et al. applied the STOPP criteria, which contain 65 criteria systematised according to the physiological systems to identify PIM [27, 28, 32]. The studies of Leikola et al. and Fu et al. used the criteria of Fick and Beer from 2003, which partly consider the diagnoses or conditions of patients [24, 29, 33]. In contrast to both tools, the PRISCUS list considers neither the physiological system nor diagnoses or conditions. Second, the lists were applied to different age groups: Bradley et al. calculated costs for pharmaceutical expenses for patients ≥70 years of age [27]. Our investigation covered prescriptions for patients ≥65 years of age. Third, different perspectives on costs were used in the studies: Cahir et al. summed the net ingredient costs, the value added tax, and the pharmacist dispensing fee [28]. In our study, costs per DDD are based on the sales data of each drug. Thus, the different approaches make a comparison of costs of PIM even more difficult.

Regarding PIM substitution, reimbursement costs are about to rise, independent of the scenario. If all prescriptions of PIM were substituted by the low-cost alternative, the total costs would be €325.9 million. Substitution by the most expensive alternative would lead to a total cost of €810.0 million. This gap can be explained by the enormous difference in the costs of the recommended therapeutic alternatives. For example, there are several different alternatives for etoricoxib (e.g., paracetamol, weak opioids (codeine, tramadol), metamizol, anticonvulsants (older anti-epileptics, newer anti-epileptics, neuromuscular blocking agents)). The cheapest alternative to etoricoxib (€1.21/DDD) is paracetamol (€0.49/DDD). The most expensive alternative is newer anti-epileptics at €4.03/DDD. The difference in costs per DDD might result in enormous cost differences in each scenario. By contrast, for solifenacin, which costs €1.43/DDD according to the PPR, the only alternative according to the PRISCUS list is trospium, with a cost of €1.05/DDD. Consequently, in both scenarios the substitution of solifenacin will decrease the total costs.

### Implication for SHI and future research

From a short-term economic perspective, there would be little incentive for SHI to support the substitution of PRISCUS agents. However, it has been shown that PIM leads to ADE which in turn can cause inpatient costs [14, 34, 35]. Furthermore, the application of PIM is directly associated with an increased risk of acute hospital admission [36–41]. Moreover, in many clinical cases there may be no adequate substitute for a PIM. In these cases, the discontinuation of the PIM without providing a substitute will be the best choice from a clinical as well as from an economical perspective. Thus, from a long-term perspective, the additional costs of physician consultation and hospitalisation due to ADEs caused by PIM might decrease and compensate for the costs of substituting the PRISCUS agents.

Our research revealed several aspects to be considered in future research on PIM. The PRISCUS list was published in 2010. Our study is based on data from 2009. Thus, our study does not analyse the effect of the PRISCUS list and possible changes in the prescription behaviour of physicians in Germany, but serves as a reference for future comparisons. Further research should investigate if and to what extent prescription patterns and PIM costs have developed due to the PRISCUS list in order to show the influence and relevance for practical use.

Our investigation demonstrated that PIM is a relevant topic in drug therapy in the elderly in Germany. The elderly account for approximately 55 % of the sales of the 30 top-selling PRISCUS agents. Similar percentages were calculated for DDDs and prescriptions. The proportion of sales, DDDs, and prescriptions to the elderly is lower in all 83 agents of the PRISCUS list. The decrease underlines that only some of the 83 PRISCUS agents are really relevant. Thus, from an economic perspective and in terms of feasibility, a shorter PRISCUS list might be more applicable for the daily health service. However, the medical perspective has to be taken into consideration as well. A short-list should also include those agents with the worst risk/benefit ratios for the elderly.

### Limitations

There are limitations to this study as a result of the methods used. First, for several PIM the recommended substitutions may not be clinically adequate in a majority of clinical cases, while in other cases the recommended substitution would lead to off-label use. For example, herbal products, such as valerian, are often named as surrogates for psycholeptics (zopiclone, zolpidem and lormetazepam). From a medical perspective, however, it is questionable if a highly effective sedative can be completely substituted by herbal alternatives. Furthermore, valerian is a non-refundable OTC and no costs occur by substituting it. Consequently, in the low-cost scenario, approximately €42 million can be "saved" by applying valerian. Second, some PRISCUS agents are only potentially inappropriate, if a certain dose is exceeded or a certain release form is applied. This was not taken into account, as the data set of WIdO consisted of total prescriptions. Third, we only analysed the 30 top-selling drugs.

Another limitation is related to our applied scenarios. In general, the scenarios assumed that a PIM would be fully substituted by the cheapest, most expensive, or mean costs. However, a complete substitution might be unlikely in daily practice, which highlights the need for a calculation based on individual patient data. In general, the proposed substitutions have to be evaluated carefully by physicians. In some cases, groups of potential surrogates include other medication form the PRISCUS list. For example, the recommended substitution of amitriptyline are SSRI. However, the group of SSRI comprises PIM as well e.g. fluoxetine. It remains unclear if in those cases PIM might be substituted by another PIM as well. For our study, we included all recommended surrogates.

## Conclusions

This is the first study to assess the economic relevance of PIM on the PRISCUS list in Germany from a SHI perspective. It was shown that PIM is present in Germany and approximately 5 % of all prescriptions made to people aged ≥65 years are potentially inappropriate. Moreover, the reimbursement costs of PIM in the elderly account for 1.36 % of total sales to patients ≥65 years of age. The substitution of PRISCUS agents would lead to an increase in costs independent of the chosen scenario. Thus, from a short-term perspective, there is no incentive for SHI to promote substitution. However, in the long-term, hospitalisation and recurrent physician visits should be addressed.

### Ethics approval and consent to participate

Not applicable.

### Consent for publication

Not applicable.

### Availability of data and materials

The datasets supporting the conclusions of this article are included within the article.

## Abbreviations

ADE: adverse drug events
AOK: German local health care fund
DDD: defined daily dose
OTC: over-the-counter
PIM: potentially inappropriate medication
PPR: pharmaceutical prescription report
SHI: statutory health insurance
WIdO: scientific institute of the German local health care fund
